# Dental Enamel Development: Proteinases and Their Enamel Matrix Substrates

**DOI:** 10.1155/2013/684607

**Published:** 2013-09-16

**Authors:** John D. Bartlett

**Affiliations:** Harvard School of Dental Medicine & Chair, Department of Mineralized Tissue Biology, The Forsyth Institute, 245 First Street, Cambridge MA 02142, USA

## Abstract

This review focuses on recent discoveries and delves in detail about what is known about each of the proteins (amelogenin, ameloblastin, and enamelin) and proteinases (matrix metalloproteinase-20 and kallikrein-related peptidase-4) that are secreted into the enamel matrix. After an overview of enamel development, this review focuses on these enamel proteins by describing their nomenclature, tissue expression, functions, proteinase activation, and proteinase substrate specificity. These proteins and their respective null mice and human mutations are also evaluated to shed light on the mechanisms that cause nonsyndromic enamel malformations termed *amelogenesis imperfecta. *Pertinent controversies are addressed. For example, do any of these proteins have a critical function in addition to their role in enamel development? Does amelogenin initiate crystallite growth, does it inhibit crystallite growth in width and thickness, or does it do neither? Detailed examination of the null mouse literature provides unmistakable clues and/or answers to these questions, and this data is thoroughly analyzed. Striking conclusions from this analysis reveal that widely held paradigms of enamel formation are inadequate. The final section of this review weaves the recent data into a plausible new mechanism by which these enamel matrix proteins support and promote enamel development.

## 1. Introduction

Tooth development is a highly orchestrated process that begins with the defined placement of individual teeth of specific shapes and sizes within the jaw. Precise signaling pathways to and from epithelial and mesenchymal cells are required for each tooth to initiate and continue along its developmental path [1, 2]. The complexity of these pathways is reflected by their high rate of incompletion. Deficiency of third molars, second premolars, and lateral incisors is common. The reported incidence of selective agenesis varies from 1.6% to 9.6% for all but third molars. Agenesis of third molars occurs in approximately 20% of the World's population [3]. Therefore, the study of tooth development has taught us how genes and tissues interact to form complex dental structures that each occupies a prespecified place within the jaw and has taught us about what can go wrong with the intricate developmental signaling pathways.

Teeth are composed of three different mineralized tissues: cementum, dentin, and enamel. Cementum is found along the tooth root and primarily serves to hold the tooth in place by binding collagen fibers (Sharpey's fibers) that are continuous with the principal fibers of the periodontal ligament. These fibers are orientated more or less perpendicularly to the cementum surface and play a major role in tooth anchorage [4]. Dentin is a bone-like matrix that forms the bulk of the tooth. It is characterized by closely packed dentinal tubules and is slightly harder than bone but softer than enamel. Dentin has an elastic quality that provides flexibility that prevents fracture of the overlying brittle enamel. Dentin and enamel are firmly bound at the dentin-enamel junction (DEJ) [4]. The enamel layer covers the crown of the tooth and is unique because it is the only epithelial derived calcified tissue in vertebrates, and it is the hardest substance in the body. Its hardness is between that of iron and carbon steel but has a higher elasticity [5]. Enamel hardness is a function of its high mineral content. Unlike bone and dentin (20–30% organic material by weight), fully formed enamel contains very little protein (less than 1% organic material) [6, 7]. Therefore, within the body, teeth are the most resistant to deterioration and have given us important anthropological clues as to how humans evolved. Although DNA analysis has taught us much about the migration patterns of our ancient ancestors, teeth have perhaps been more important in identifying our ancestor's food preferences and lifestyles. Their shape, wear patterns, and carbon isotope compositions are unique indicators of our past behaviors [8].

## 2. Overview of Enamel Development

This review focuses on enamel proteinases, their substrates, and on recent discoveries that help us to better understand the process of enamel development. For outstanding review articles about enamel development prior to 1999, please see the following reviews: [9–11]. The review by Simmer and Fincham [9] provides an excellent historical perspective about enamel development, the review by Smith [11] provides a detailed examination on how ions are controlled during enamel formation, and the review by Bartlett and Simmer [10] focuses on what was known about the enamel proteins and proteinases prior to 1999.

**Figure 1:**
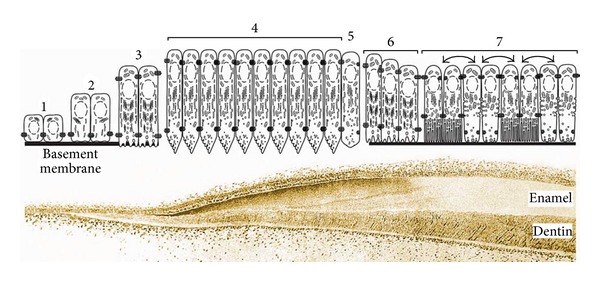
Ameloblast changes during enamel formation. The epithelial cells of the inner enamel epithelium (1) rest on a basement membrane containing laminin. These cells increase in length and become differentiating ameloblasts above the predentin matrix (2). Presecretory ameloblasts send processes through the degenerating basement membrane as they initiate the secretion of enamel proteins on the villous surface of mineralizing dentin (3). After establishing the dentin-enamel junction and mineralizing a thin layer of aprismatic enamel, secretory ameloblasts develop a secretory specialization or Tomes' process. Along the secretory face of the Tomes' process, in place of the absent basement membrane, secretory ameloblasts secrete proteins at a mineralization front where the enamel crystals grow in length (4). Each enamel rod follows a retreating Tomes' process from a single ameloblast. At the end of the secretory stage, ameloblasts lose their Tomes' process and produce a thin layer of aprismatic enamel (5). At this point, the enamel has achieved its final thickness. During the transition stage, the ameloblasts undergo a major restructuring that diminishes their secretory activity and changes the types of proteins secreted (6). KLK4 is secreted, which degrades the accumulated protein matrix. During the maturation stage ameloblasts modulate between ruffled and smooth-ended phases (7). Their activities harden the enamel layer. The histology of the developing tooth was adapted from The histology of the developing tooth was adapted from Uchida et al. Arch Histol Cytol 54:527-538, 1991 and the schematic plus tooth was published in: Hu et al., Cells Tissues Organs 186:78-85, 2007. DOI: 10.1159/000102683.

Enamel development (amelogenesis) can be broken down into four defined stages: presecretory, secretory, transition and maturation. The stages are defined by the morphology and function of the ameloblasts (Figure 1). The ameloblasts are a single cell layer that covers the developing enamel and is responsible for enamel composition. Ameloblasts are part of the enamel organ that is composed of an outer epithelial layer, the stellate reticulum, the stratum intermedium, and the inner enamel epithelium (ameloblast layer). The basal (proximal) end of the preameloblast is attached by desmosomes to the stratum intermedium, and the apical (distal) end is attached by hemidesmosomes to a basement membrane (basal lamina) located at the future site of the DEJ.

### 2.1. Organization of the Enamel Organ

The outer enamel epithelium is a single layer of cells that covers the enamel organ and is contiguous at the cervical loop with the ameloblasts (inner enamel epithelium) located initially at the future DEJ. The cells of the stellate reticulum are sandwiched between the outer dental epithelium and stratum intermedium and secrete hydrophilic glycosaminoglycans into the extracellular compartment. This causes water to diffuse into the enamel organ which in turn forces these cells apart. Since these cells are all interconnected by desmosomes, they are stretched into a star shape and are therefore termed the stellate reticulum [4]. The stratum intermedium forms a boundary between the stellate reticulum and the inner enamel epithelium and may be important for shuttling ions to and from the ameloblasts [19]. The ameloblasts are responsible for secreting enamel matrix proteins and proteinases, inducing mineral ribbons to form, and organizing them into rod and interrod patterns typical for each vertebrate species.

### 2.2. Presecretory Stage

One of the earliest events occurring during the presecretory stage, just prior to mineral formation, is the deposition of predentin by odontoblasts at the future DEJ [20]. This occurs first at the cusp tips and progresses to the cervical regions of a tooth. Predentin is composed primarily of collagen but also contains noncollagenous proteins. Predentin is the first to mineralize [21] starting slightly under what will become the DEJ. The dentin becomes thicker as the wave of mineralization moves latterly and away from the DEJ towards the future pulp chamber. This decreases the size of the chamber as mineralization proceeds. However, almost immediately after initial dentin mineralization near the DEJ, differentiating preameloblasts extend cytoplasmic projections through the basement membrane that remove and destroy it, and then the ameloblasts begin secreting enamel matrix proteins which rapidly initiate mineralization [4].

### 2.3. Secretory Stage

The preameloblasts transform into secretory stage ameloblasts by elongating into tall columnar cells and by forming Tomes' processes at their apical ends nearest the forming enamel. The Tomes' process is a conical structure that points toward the forming enamel matrix. Enamel matrix proteins are primarily secreted from one side of the Tomes' process (secretory face), and all ameloblasts within a row secrete protein from the same side of their Tomes' processes. The first enamel crystals (ribbons) formed grow between the dentin crystals perhaps by mineralizing around dentin proteins such as collagen. These crystal ribbons elongate at the mineralization front where enamel proteins are secreted [22]. Secretory stage enamel is protein rich and has a soft cheese-like consistency.

The ameloblasts start secreting large amounts of enamel matrix proteins as they move away from the dentin surface so that the nascent enamel layer can thicken. In association with newly secreted proteins, long thin mineral ribbons form rapidly normal to the secretory surface of the ameloblasts. The parallel crystallite ribbons, approximately 10,000 to 40,000 [23], will eventually form into a rod (prism) and each ameloblast is responsible for creating one enamel prism all of which collectively form a highly ordered 3D structure. Soon after the initial formation of crystallite ribbons, the ameloblasts develop their apical Tomes' processes. This establishes a two-compartmental system where proteins destined to form interrod enamel tend to exit near the “base” of the process, whereas those involved in rod formation tend to exit from the “tip” (secretory face) of the process. During this time, protein cleavage products are either reabsorbed by the ameloblasts or may accumulate between the rod and interrod enamel. Mineral crystallites forming within the rod will grow progressively in *c*-axis length parallel to one another as ameloblasts move away from the dentin surface. Mineral crystallites developing between the rods (interrod) may have more limited lengths, but they are always positioned spatially to be at angles relative to rod crystallites [4]. 

During the secretory stage, ameloblasts not only move away from the dentin as the enamel thickens, but they also move in groups that slide by one another, and this movement culminates in the characteristic decussating enamel prism pattern observed in rodent incisors [24] or the entwined gnarled prism pattern seen in human molars [25]. As this occurs, ameloblasts secrete four different proteins into the enamel matrix. Three are presumed structural proteins and one is a proteinase. The structural proteins are amelogenin (AMELX), ameloblastin (AMBN), and enamelin (ENAM), and the proteinase is matrix metalloproteinase-20 (MMP20, enamelysin). Amelogenin comprises approximately 80–90% of the organic matter within the secretory stage enamel matrix, and ameloblastin and enamelin comprise roughly 5% and 3–5%, respectively [13]. MMP20 is present in trace amounts. The precise function of these proteins remains unclear. However, human mutations in *AMELX, ENAM*, and *MMP20* genes and mouse knock-out models have provided striking clues that have dramatically changed the way we envision amelogenesis, and this has occurred within a span of less than two decades. For example, as will be described in detail below, enamel crystals will form in the absence of amelogenin but will not form if either ameloblastin or enamelin is absent. By the end of the secretory stage, the enamel layer has achieved its full thickness. It is not until the end of the maturation stage when the proteins are almost completely removed, that the enamel achieves its final hardened form.

### 2.4. Transition and Maturation Stages

The beginning of the transition stage is dependent on the species and on the specific developing tooth. However, prior to when the enamel layer reaches its full thickness, the ameloblasts no longer move relative to each other. They retract their Tomes' processes, smooth off the enamel surface with a final coating of aprismatic enamel, transition (transition stage) into shorter and fatter maturation stage cells, and, reapply a new basal lamina and the ameloblasts start modulating between ruffle and smooth-ended cells at the enamel surface [11]. It is during the maturation stage that ameloblasts actively secrete kallikrein-related peptidase-4 (KLK4) to help remove the mass of previously secreted and partially hydrolyzed matrix proteins from the enamel layer so that the rod and interrod crystallites can expand in volume to occupy as much space as possible within the enamel layer.

Enamel mineral is very similar to hydroxyapatite [Ca_5_OH (PO_4_)_3_] but also contains low percentages of carbonate, sodium, and magnesium. The initial enamel ribbons are only a few apatitic unit cells in thickness (about 10 nm) with a width of approximately 30 nm [26, 27] and a length that may extend through the entire thickness of the enamel layer. After the enamel rods have formed, an area exists in some species between the rod and interrod enamel that contains a thin organic matrix with no crystals [28–30]. This structure is often called the rod sheath or sheath space and is prominent in humans [31].

### 2.5. Summary

Enamel development can be broken down into four defined stages. During the presecretory stage, the ameloblasts poke through and remove the basal lamina and start secreting enamel matrix proteins at the forming DEJ. Soon after, as the ameloblasts enter the secretory stage, they elongate, develop Tomes' processes, and secrete large amounts of proteins into the enamel matrix which are necessary for the enamel crystallite ribbons to form and lengthen. Once the enamel reaches full thickness, the ameloblasts transition into shorter protein reabsorbing cells that define the maturation stage of development and at the end of this stage the enamel will achieve its final hardened form. These general features of amelogenesis are remarkably constant in different species [32].

## 3. Enamel Proteinases

Approximately 50 years ago, it was demonstrated that developing enamel had a high protein content, whereas mature enamel did not [33, 34]. It was discovered that the enamel matrix proteins were removed during the maturation stage of amelogenesis [35]. Studies on developing bovine enamel demonstrated that the percentage of protein by weight dropped from 30% during the secretory stage to 2% during the early maturation stage [36]. In the rat incisor, a similar decline was associated with a significant change in the amino acid composition of total enamel matrix proteins [37]. Thus, a role for proteinases in the degradation and export of enamel proteins was advanced. Approximately 25 years ago, several investigations suggested that as the forming enamel passes through the secretory stage and into the maturation stage of development, the enamel proteinases undergo a change in profile. This change was first identified by Overall and Limeback [38] who used enzyme inhibitors to demonstrate that metalloproteinase(s) were present during early enamel development and that serine proteinase(s) were present during the later stages. Although there was some overlap, this change in enzyme profile was confirmed by several other investigators [39–43]. Therefore, prior to identification of any specific protease within developing dental enamel, evidence suggested that at least two classes of enzymes were present. A proteinase of the metalloproteinase class was present early during the secretory stage, and a proteinase of the serine class was present late during the maturation stage of enamel development. 

The proteinase expressed during the secretory through early maturation stage is MMP20 [44] and the proteinase expressed from the transition through maturation stages is KLK4 [45]. To date, these are the only two proteinases known to be secreted into the enamel matrix. Both proteinases are present in trace amounts during enamel development, and each proteinase was separately cloned by performing PCR-based homology cloning [46, 47]. KLK4 was originally named enamel matrix serine proteinase-1 (EMSP1), but its name was changed to kallikrein-4-related peptidase-4 because the gene encoding KLK4 locates in the kallikrein gene cluster. 

## 4. Matrix Metalloproteinase-20/Enamelysin

### 4.1. MMP20 Nomenclature

MMP20 was originally cloned from a porcine enamel organ cDNA library [46]. Although it has since been shown to be expressed in odontoblasts of the pulp organ [48], originally its expression was thought to be confined to the enamel organ. This novel MMP was therefore named “enamelysin”. At the 1997 Gordon Research Conference on Matrix Metalloproteinases, a group headed by Dr. J. Fredrick Woessner designated this novel MMP as “MMP20”, and this designation was first published with the cloning of the human *MMP20* mRNA [49]. 

### 4.2. MMP20 Localization

MMP20 was cloned by PCR-based homology cloning from pig enamel organ, and supporting northern blots demonstrated its tissue restricted pattern of expression. Therefore, it remained uncertain as to which cells of the enamel organ expressed MMP20, and whether MMP20 was secreted into the enamel matrix. These issues were resolved by a report that used immunogold labeling analysis to identify MMP20 location and used a unique, zymography technique that started with a gel overloaded with porcine enamel matrix proteins. The zymogram was incubated for a short time to demonstrate proteolysis of the copolymerized substrate and was then subjected to western blotting to demonstrate that those zones of proteolysis were attributed to MMP20 [48]. Another paper showed *in situ *hybridization to demonstrate that both ameloblasts and odontoblasts express *Mmp20 *transcripts [44]. Thus, MMP20 became the first proteinase to be definitively identified as expressed by the ameloblasts of the enamel organ and was identified by name as the first proteinase secreted into the developing enamel matrix. 

### 4.3. MMP20 Tissue Expression

MMP20 has a highly restricted pattern of expression. Very few tissues or cell lines express MMP20. Reverse-transcription-PCR was used to detect various MMP expression in 51 different cell lines. However, none of the cell lines tested positive for MMP20 [50]. In contrast, MMP20 was expressed in a few pathologic tissues such as in ghost cells of calcifying odontogenic cysts [51], odontogenic tumors [52], human tongue carcinoma cells [53], and in bradykinin treated granulosa cells isolated from the follicles of porcine ovaries [54]. No recent reports have observed MMP20 expression in any tested cell line (reviewed in [55]). 

Our laboratory assessed *Mmp20* expression by quantitative-real time PCR (qPCR) of mRNA isolated from various mouse tissues. We found that except for developing teeth, *Mmp20* was only expressed at very low levels in the large intestine. MMP20 expression in the intestine was too low to be detectable by northern blotting and was approximately 5000 times lower than the levels observed in 4-day-old tooth buds. *Mmp20* was not expressed in small intestine, brain, heart, kidney, liver, lung, pancreas, spleen, or stomach [56]. Incomprehensively, in 2009, one group published a report stating that an SNP within *MMP20 *was associated with kidney aging. Two years later, the same group published a review article reiterating their finding. An *MMP20* association with kidney aging has not been confirmed by any other group and no other papers have been published on this subject. The finding was problematic because *MMP20* is not expressed in the kidney. An NCBI search for “MMP20” in the UniGene search engine reveals that not a single *MMP20* EST has been recovered from the kidney. Of even greater consequence, it was discovered that in Baleen whales, *Mmp20* is a pseudogene. Baleen whales lack teeth and since nonfunctional *Mmp20* genes are found only in mammals lacking enamel, the authors postulated that the only nonoverlapping essential function of MMP20 is in dental enamel formation [57]. Therefore, to date, MMP20 is considered a tooth-specific MMP.

### 4.4. MMP20 Activation

How MMP20 becomes activated remains an enigma. Two MMP20 bands of approximately 46 and 41 kDA are observed on immunoblots and zymograms [48]. Normally one would consider the upper band to be pro-MMP20 and the lower band to be active MMP20 with its propeptide cleaved. However, The identity of the 46 and 41 kDa forms of purified porcine MMP20 was assessed by performing immunoblots, zymography, reverse-phase HPLC, and protein sequencing after exposure to oxidizing and reducing conditions [58]. The oxidizing and reducing conditions were performed to leave intact (oxidizing) or release (reducing) the disulfide bond that connects the first and last amino acids of the C-terminal hemopexin domain. When the disulfide bond was left intact both the 46 and 41 kDa forms of MMP20 were observed. When the disulfide bond was released, the 41 kDa band was replaced by a catalytically active 27 kDa band. Edman sequencing of the three bands showed they all contained the catalytic domain at their N-termini (YRLFPGEPK), proving that none of the bands corresponded to the MMP20 zymogen. In addition, under reducing conditions that release the disulfide bond, a 17 kDa protein band stained positive for MMP20 on the immunoblot, and its N-terminal sequence started with Ile^336^ of the hemopexin domain. In aggregate, these observations demonstrate that one of the 46 or 41 kDa MMP20 bands is the active intact protease, while the other band is active protease that has been cleaved in the hemopexin domain after Thr^335^ [58]. This C-terminal peptide is covalently attached by the disulfide bridge, and when that bridge is released, a portion of the cleaved hemopexin domain falls away to generate the 27 kDa catalytic domain and the 17 kDa hemopexin domain. The MMP20 zymogen is not often observed within the secretory stage enamel matrix, presumably due to its efficient activation *in vivo* [58] and/or because the demineralization procedures necessary to extract MMP20 from the matrix may activate the zymogen. 

The MMP20 propeptide does not contain an RXXR furin consensus sequence that allows activation in the trans-Golgi network. However, recombinant MMP20 autoactivates [49] and appears to readily remove its hemopexin-like domain to form a catalytically active species of approximately 22–27 kDa [59–61]. Therefore, MMP20 was postulated to autoactivate *in vivo*. Additionally, membrane-type MMP1 (MT1-MMP, MMP14) has a transmembrane domain and binds to cell membranes with its catalytic domain located extracellularly. MMP14 was identified on the cell surface of ameloblasts and odontoblasts of the developing tooth [62], and MMP14 does activate the MMP20 zymogen [63]. So, this is also a possible means of activation *in vivo*. To date, no strong evidence exists on how MMP20 becomes activated within the enamel matrix. 

### 4.5. MMP20 Substrate Specificity

Soon after its discovery, MMP20 was shown to cleave the most abundant enamel matrix protein amelogenin [49]. Since that time, MMP20 substrate specificity was characterized by use of an iterative mixture-based random dodecamer peptide library screen with Edman sequencing of MMP20 cleavage products. MMP20 was found to have a broad substrate specificity with a deep and wide catalytic pocket that can accommodate substrates with large aromatic residues in the P1′ position. As is typical for MMPs, MMP20 is highly selective for hydrophobic residues at the P1′ position, and its preferred amino acid at this position is leucine with methionine and tyrosine also being strongly selected. MMP20 was relatively nonselective at the P2′ position and had a slight preference for smaller residues at the P3′ position which had been previously observed in a few other MMPs. Alanine and proline are preferred amino acids at the P3 position. This study suggested that MMP20 expression may be restricted to tooth tissues because of its broad substrate specificity that might otherwise cause tissue destruction if expressed elsewhere [56]. This conclusion is reinforced by the fact that the MMP20 zymogen is rarely observed *in vivo*. 

MMP20 has been well characterized for its ability to cleave the most abundant enamel matrix protein, amelogenin [48, 49, 60, 61, 64–66]. Two different studies identified the exact MMP20 cleavage sites in amelogenin. The first used recombinant MMP20 and amelogenin [61], and for the second study, the authors used their considerable protein purification expertise to purify native amelogenin and native MMP20 from developing pig teeth and also used quenched fluorescent peptides to confirm their cleavage site results [66]. The precise cleavage sites were identified by various means including mass spectrometry and protein sequencing. These cleavage sites were then compared to previously identified amelogenin cleavage products isolated from extracted porcine enamel. All of the MMP20 amelogenin cleavage sites generated *in vitro *were also identified from amelogenins extracted from normal porcine enamel. Therefore, since no other amelogenin cleavage products were identified in secretory stage porcine enamel other than those generated by MMP20, it was concluded that MMP20 is likely the only proteinase present in the enamel matrix during the secretory stage of enamel development [61, 66]. 

The same group assessed the other structural enamel matrix proteins (ameloblastin, enamelin) to determine if they were MMP20 substrates. Similar exacting protocols used to identify amelogenin cleavage products were also used to identify ameloblastin and enamelin cleavage products. As was observed for amelogenin, all of the ameloblastin MMP20 cleavage sites identified *in vitro* were also identified from ameloblastin extracted from porcine enamel *in vivo* [67, 68]. It is difficult to identify enamelin cleavage sites because enamelin is so quickly cleaved within the enamel matrix. Enamelin has a 186 kDa apparent molecular weight and is highly glycosylated. However, only a 32 kDa enamelin cleavage product accumulates within the maturing subsurface enamel layer [69]. MMP20 does not cleave the glycosylated 32 kDa enamelin [70]. Therefore, based in part on the amelogenin and ameloblastin MMP20 cleavage results, it was concluded that MMP20 is likely responsible for generating the 32 kDa enamelin cleavage product *in vivo*. MMP20 will also cleave the KLK4 propeptide to produce catalytically active KLK4 [71]. In addition, MMP20 is expressed in the odontoblasts of the pulp organ as is MMP2 and both MMP20 and MMP2, were each demonstrated to cleave dentin sialophosphoprotein, which is the major noncollagen secretory product of odontoblasts responsible for dentin formation [72]. Therefore, the first evidence suggesting that MMP20 plays an critical role in enamel development was with the first report demonstrating that all amelogenin cleavage products observed *in vivo* are expected MMP20 cleavage products [61].

In addition to enamel and dentin proteins, MMP20 was also demonstrated to cleave E-cadherin [73], casein and/or gelatin [48, 53, 59], aggrecan and cartilage oligomeric matrix protein [74], type V collagen [56], type XVIII collagen [75], fibronectin, type IV collagen, tenascin-C, and laminin-1 and -5 but not type I or type II collagen [53]. These reports confirm the broad substrate specificity of MMP20 and lend credence to the theory that MMP20 has a highly restricted pattern of expression because its expression elsewhere could cause tissue damage (reviewed in [55]).

### 4.6. The Mmp20 Null Mouse

**Figure 2:**
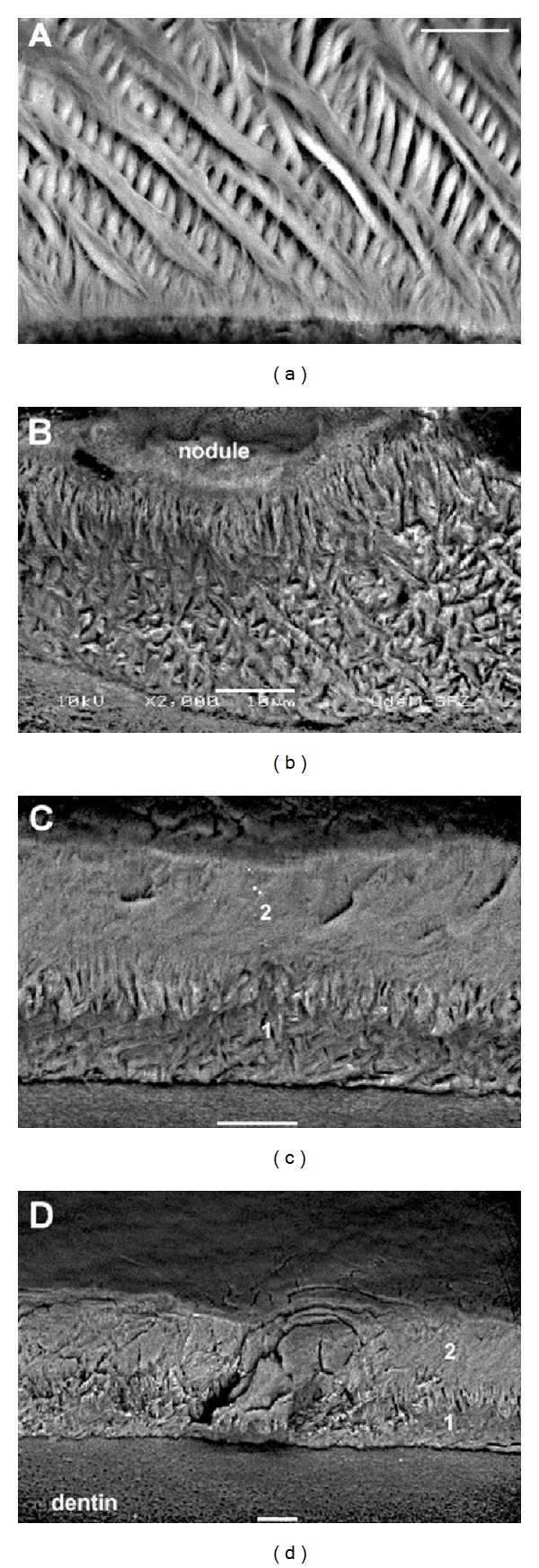
Enamel rod patterns of mandibular incisors from wild-type and *Mmp20* null mice. The wild-type enamel had crisscrossing (decussating) rows of enamel rods (a). The *Mmp20 *null enamel may have a poorly organized rod pattern (b), no rod pattern with a poorly organized rod layer beneath (c), or virtually no rod pattern whatsoever (d). 1 and 2 designate the two different enamel layers. All magnification bars are 10 *μ*m in length. This figure was originally published in: Bartlett et al. Eur J Oral Sci. 119 (Suppl 1): 199-205, 2011. D01:10.1111/j.1600-0722.2011.00864.x.

The MMP20 preproenzyme is composed of 483 amino acids, while the proenzyme has 461 residues and the active form has 376 amino acids [64]. The mouse *Mmp20* gene consists of 10 exons (all coding) spanning approximately 65 kb within the MMP gene cluster at the centromeric end of chromosome 9 [76]. The *Mmp20* null mouse was engineered by deleting the majority of exon 4 and exon 5 [77]. Exon 5 encodes the highly conserved zinc binding site (HEXGHXXGXXH) present in the catalytic domain of MMP family members. This deletion rendered MMP20 catalytically inactive. The *Mmp20* null mouse was demonstrated to not process amelogenin properly, had an altered enamel protein and enamel rod pattern, had hypoplastic (thin) enamel (Figure 2), had enamel that broke off from the dentin, and had a deteriorating enamel and enamel organ morphology as enamel development progressed [77]. A subsequent study showed that the weight percent of *Mmp20* null mouse mature enamel was 7–16% less than that of wild-type controls and that the overall enamel mineral was reduced by 50% and enamel hardness was decreased by 37%. Remarkably, the biggest difference in mineral content between the *Mmp20* null and controls occurred in the nearly mature enamel when *Mmp20* was normally no longer expressed [78]. This suggested that MMP20 acts directly or indirectly to facilitate the removal of maturation stage enamel matrix proteins. 

Recent reports examining the *Mmp20* ablated mice have suggested that MMP20 does something else besides cleaving enamel matrix proteins. For example, Tomes' processes are normally formed after ameloblasts have formed an initial thin layer of mineralized aprismatic enamel at the DEJ. These Tomes' processes are later retracted permanently just prior to when the ameloblasts produce the final thin layer of mineralized aprismatic enamel at the outer enamel surface. This is when ameloblasts start their transition into the maturation stage. However, *Mmp20* null ameloblasts abnormally extend, retract, and later reextend their Tomes' processes during enamel development [73]. This suggests that the signaling mechanism responsible for developmental progression to the maturation stage is deficient in the null mice. So, how could MMP20 play a role in ameloblast cell signaling? It was proposed that MMP20 does this by cleaving the extracellular domains of cadherins that are part of the adherens junction (AJ) complex responsible for ameloblast cell-cell adhesion [19, 73]. Cadherins are transmembrane proteins where the extracellular domains connect through homotypic transpairing between cadherins on adjacent cells and the intracellular domains are linked to the actin cytoskeleton by catenins (reviewed in [79]). Ameloblasts express E-, N-, and P-cadherins, *β*-catenin, and p120-catenin during dental enamel development [80–86]. A major pathway for signal transduction by AJs involves regulation of *β*-catenin and p120-catenin, which can act as either structural proteins at cell-cell junctions or as transcription factors in the cell nucleus (reviewed in [87]). When MMPs cleave a cadherin's extracellular domain, *β*-catenin and p120-catenin are removed from their position near the cell membrane and, under certain circumstances, will translocate to the cell nucleus thereby promoting cell migration, cell invasion, and/or cell proliferation [88–92]. E-cadherin is among the cadherins expressed by ameloblasts [19], and MMP20 was shown to cleave the E-cadherin extracellular domain [73]. Therefore, a possible way that MMP20 could play a role in ameloblast cell signaling is by cleaving cadherin extracellular domains on ameloblasts that in turn release *β*-catenin and p120-catenin from their disassembled intracellular domains. These released catenins would then be transported to the ameloblast nucleus where they would participate in cell signaling. It was definitively demonstrated that *β*-catenin, p120-catenin, and cadherins are essential for tooth and enamel development [80, 93] and that MMP20 cleaves the E-cadherin extracellular domain *in vitro *[73]. However, it remains to be determined if MMP20 actually cleaves cadherins *in vivo *to initiate ameloblast cell movement and/or a signaling cascade. 

The enamel from *Mmp20* ablated mice has striking features. Normally a thin, highly mineralized initial layer of enamel begins forming during the secretory stage at the dentin-enamel junction. However, this does not occur in *Mmp20 *null mice and this may be a primary reason why the enamel from these mice, sheers off the dentin. In addition, the fully developed null mouse enamel appears histologically as two distinct layers, and the enamel surface is marred by calcified nodules that vary greatly in their dimensions [14, 94, 95]. Neither layer resembles wild-type enamel nor does either layer have the characteristic rod/interrod organization. The inner layer closest to the dentin appears homogenous, is not well mineralized, and does not vary greatly in thickness. However, the outer layer closest to the ameloblasts shows large variations in thickness, and large nodules can be observed protruding from this layer. It is not known why the *Mmp20* null mouse enamel forms in this manner. It was proposed that during the secretory stage, a 25–30 *μ*m defective mineral layer is deposited on top of the dental-enamel junction that contains abundant uncleaved enamel proteins and that during the maturation stage, ions that normally contribute to the maturation of the crystallites cannot penetrate the inner enamel layer and instead precipitate as a second layer on top of the first [95]. The reasons for why the calcified nodules form are equally perplexing. The *Mmp20* null mouse ameloblasts do cover the nodules, but it remains uncertain as to whether the ameloblasts “ball up” prior to nodule formation or if they acquire their dysplastic shape because the nodules form first. KLK4 is expressed and is active in *Mmp20* ablated mice. However, unabsorbed protein can be observed in the maturation stage at the ameloblast-enamel interface. It was therefore proposed that these proteins may promote ectopic nodule calcification [14]. This proposal supports the notion that the nodules form first and disrupt the normally smooth ameloblast layer. Taken together, the *Mmp20 *ablated mice have taught us much about the function of MMP20 and enamel formation, but we still have much more to learn.

### 4.7. Human MMP20 Mutations

Human MMP20 is expressed from a gene on chromosome 11q22-q23 that has 10 exons (all coding). The MMP20 protein has 483 amino acids, and its domain structure includes a signal peptide necessary for MMP20 secretion, a propeptide that maintains enzyme latency, a catalytic domain with a zinc binding site, and a hinge domain that links the catalytic domain to the C-terminal hemopexin-like domain [49]. Its only posttranslational modification is a disulfide bridge connecting the first and last amino acids of the hemopexin domain [58]. Although MMP20 is not glycosylated as are some other MMPs, MMP20 does share the characteristic domain structure found in most other MMP family members. 

Inherited enamel defects that occur in the absence of a generalized syndrome are collectively designated as *amelogenesis imperfecta* (AI). AI can be inherited by autosomal dominant (ADAI), autosomal recessive (ARAI), and X-linked modes of transmission. Classification of AI can be divided into fourteen distinct subtypes based on clinical phenotype and mode of inheritance [96]. However, this can be narrowed down to three main types. These are hypoplastic, hypomaturation, and hypocalcified AI. Hypoplastic enamel is thin and is associated with defective matrix synthesis that occurs as the enamel increases in thickness. Hypomaturation enamel is soft and typically stained but is of normal thickness and is associated with a failure to remove enamel matrix proteins. Hypocalcified enamel is the most severe and appears to represent a more fundamental disturbance that affects both early and late stage enamel development. Hypocalcified enamel is typically soft, rough and is rapidly lost by attrition (reviewed in [97]). 

Seven different human *MMP20* mutations are known to cause autosomal recessive hypomaturation or hypoplastic-hypomaturation *amelogenesis imperfecta.* Five of these mutations cause pigmented hypomaturation AI [98–101], and two resulted in hypoplastic-hypomaturation AI [102, 103]. In all seven cases, the teeth are normal in size, but the enamel layer does not contrast well with dentin on radiographs, and the enamel tends to chip away from the underlying dentin. One of the hypoplastic-hypomaturation phenotypes has enamel with surface roughness and a yellowish-brown pigmentation that is present during tooth eruption, suggesting that the staining is intrinsic and not acquired [102]. Six of the seven *MMP20* mutations known to cause AI are homozygous mutations, and one is a compound heterozygous mutation. The homozygous *MMP20* mutations include, in order of publication date, mutations in the intron 6 spice acceptor (IVS6-2A-T) that likely causes the mRNA to be degraded by nonsense mediated decay, a missense mutation in the conserved active site residue (p.His226Gln) of the catalytic domain that eliminates enzyme activity, a premature stop codon in the propeptide (p.Trp34X), a mutation in a highly conserved residue present in the hemopexin domain (p.Ala304Thr) that likely causes misfolding with resulting endoplasmic reticulum-associated degradation, a missense mutation in the conserved active site residue (p.His204Arg) of the catalytic domain that coordinates the structural zinc ion, and a missense mutation in an invariant residue (p.Thr130Ile) present in the catalytic domain. The compound heterozygous mutation has one allele with the just described p.Thr130Ile mutation, and the other allele has a nucleotide deletion leading to a premature stop codon (p.Asn120fz*2). Other than defective tooth enamel, no other phenotype is observed in these patients. Therefore, genetic mutations in both mice and humans and the lack of functional MMP20 in mammalian species without enamel (baleen whales) demonstrate that MMP20 is essential for enamel formation but is not essential for any other biological function.

## 5. Kallikrein-Related Peptidase-4

### 5.1. KLK4 Nomenclature

 KLK4 was originally named enamel matrix serine proteinase-1 (EMSP1) [47]. Later, it was found in normal and neoplastic prostate epithelial tissues and was named “prostase” [104]. Another group named it kallikrein-like proteinase-1 (KLK-L1) [105]. Finally, the Human Gene Nomenclature Committee (London, UK) adopted the tissue neutral term “serine proteinase 17” (PRSS17). However, this designation was later deemed unsatisfactory, and the official designation was changed to kallikrein-4 (KLK4). It was so named because KLK4 is the fourth member of a cluster of 15 serine protease genes that comprise the human kallikrein locus near the telomere on the long arm of chromosome 19. However, even this name required further refinement by the Nomenclature Committee. Now the KLK4 designation refers to “kallikrein-related peptidase 4”. Unfortunately, a PubMed search using the search term “KLK4” will not retrieve the original publication describing the first cloning of KLK4/EMSP1 [47] or other papers using early designations.

### 5.2. KLK4 Localization

In 1977, a protease was purified from pig enamel [106] that was later demonstrated to be inhibited by the serine proteinase inhibitors phenylmethylsulfonyl fluoride (PMSF) and diisopropylfluorophosphate (DIFP) [107]. This protease was expressed during the early maturation stage when the enamel proteins are reabsorbed from the hardening enamel [38]. Like MMP20, KLK4 was cloned by PCR-based homology cloning from porcine cDNA with subsequent screening of a porcine cDNA library. This was accomplished by one team of investigators. However unlike MMP20, it was already known that KLK4 was secreted into the enamel matrix because another team of investigators had been purifying KLK4 protein from 2,000 unerupted pig incisors for protein sequencing and eventual cloning. At first, neither team knew of each other's KLK4 research. However, the epiphany occurred at a Gordon Research Conference when the principle investigator (Bartlett) from one team displayed a KLK4 poster that was directly adjacent to the KLK4 poster from one of the principle investigators (Simmer) from the other team. After careful consideration of the avenues to pursue, we decided to collaborate [47] and our collaborative research has continued ever since. Thus, KLK4/EMSP1 became the second proteinase identified by name that is secreted into the developing enamel matrix.

### 5.3. KLK4 Tissue Expression

KLK4 is a glycosylated, chymotrypsin-like serine protease that is expressed and secreted by transition to maturation stage ameloblasts [45, 108, 109]. KLK4 protein has not been isolated from any tissue, other than from developing teeth [66, 71]. However, several studies have performed immunoassays or qPCR techniques to identify KLK4 in various tissues and many of these studies conflict with one another as to exactly where KLK4 is expressed (reviewed in [110]). All prior KLK expression studies in nondental tissues (excluding cancers) were performed in adult mice. Clarity from these convoluted data was made possible by the development of a gene-targeted mouse strain that has a *LacZ* reporter gene with a mouse nuclear localization signal (NLS-**β**gal) inserted at the natural *Klk4* translation initiation site which can be used to assay *Klk4* expression using **β**-galactosidase histochemistry [15]. So, the *Klk4* knock-out/*LacZ* knock-in mice were used to identify the tissues expressing *Klk4. *The tissues tested were teeth, adult prostrate, liver, kidneys, submandibular salivary glands, ovaries, testes, vas deferens, and epididymis. The results demonstrated that the expression of KLK4 by maturation stage ameloblasts was far stronger than that of any soft tissue tested. In the adult organs surveyed, the striated ducts of the submandibular salivary gland and small patches of prostate epithelia were the only sites that showed unambiguous KLK4 expression. Furthermore, no obvious morphological abnormalities were observed in any of the nondental tissues examined suggesting that their normal development is not *Klk4* dependent [110]. As is true for MMP20, it appears that the only essential, nonoverlapping function of KLK4 is in enamel development. 

### 5.4. KLK4 Activation

It remains uncertain how KLK4 is activated *in vivo.* Active KLK4 has a predicted molecular weight of 24 kDa, but this value does not take into account posttranslational modifications. Mouse and pig KLK4 each have three Asn residues in the appropriate context for glycosylation, while human KLK4 has just one [111]. The KLK4 zymogen has not been observed in the enamel matrix (J. P. Simmer, personal communication). So, as for MMP20, it is likely that mostly active KLK4 resides in the maturing enamel. Removal of the KLK4 propeptide is essential for activation because it allows a salt linkage to form between the new N-terminus and the side chain of Asp194, and this is essential for enzyme activity [112]. Unlike the other kallikrein-related peptidases, KLK4 has a Gln as the last residue of its propeptide and not an Arg or Lys which means that KLK4 cannot be activated by trypsin-like enzymes [113]. KLK4 cannot activate itself but can be activated by MMP20 and thermolysin *in vitro *[71]. However, KLK4 is active in *Mmp20* ablated mice [114], so MMP20 cannot be the sole KLK4 activator. Although it has not been directly demonstrated, perhaps the best candidate for the activation of KLK4 *in vivo* is dipeptidyl peptidase I (Cathepsin C, CTSC). CTSC activates KLK4 *in vitro* and is almost ubiquitously expressed. In the enamel organ, CTSC is expressed at progressively increasing levels as development progresses to the early maturation stage when KLK4 begins its expression. Furthermore, this same study demonstrated that enamel from CTSC null mice was significantly softer than enamel from wild-type controls [115]. Therefore, it remains a possibility that this cysteine aminopeptidase is the primary enzyme that activates KLK4.

### 5.5. KLK4 Substrate Specificity

The first report demonstrating that KLK4 cleaves amelogenin used native porcine KLK4 incubated with recombinant pig amelogenin, and this resulted in the generation of twelve cleavage products which were characterized by N-terminal sequencing [71]. It was subsequently demonstrated that the primary MMP20 N-terminal cleavage product, tyrosine-rich amelogenin polypeptide (TRAP), was further cleaved by KLK4 which was consistent with the notion that KLK4 cleaves enamel matrix proteins into small peptides to facilitate their export out of the enamel as the enamel hardens [66]. Porcine ameloblastin was stably expressed and secreted from HEK293-N cells and was purified for digestion by KLK4. The cleavage products were characterized by N-terminal sequencing, and KLK4 was shown to cleave ameloblastin at nine different sites [68]. The 32 kDa enamelin is presumed to be an MMP20 cleavage product, and it is the only domain of the parent protein that accumulates in the deeper, more mature enamel layer. Native porcine KLK4 was incubated with native porcine 32 kDa enamelin, and the digestion products were fractionated by reverse-phase high-performance liquid chromatography (RP-HPLC) and characterized by Edman sequencing, amino acid analysis, and mass spectrometry. KLK4 digestion of the 32-kDa enamelin generated nine major cleavage products [70]. Therefore, KLK4 cleaves all the structural enamel matrix proteins that are known to be secreted into the enamel matrix, and recent evidence suggests that KLK4 may also hydrolyze MMP20. This is because in *Klk4* ablated mice, MMP20 is active well into the maturation stage when MMP20 activity has normally ceased [114]. 

KLK4 was assessed for its substrate specificity by using recombinant KLK4 to screen tetrapeptide positional scanning synthetic combinatorial libraries (PS-SCL). The identified preferred P1-P4 positions were P1-Arg; P2-Gln/Leu/Val; P3-Gln/Ser/Val, and P4-Ile/Val. Based on these results a database search for substrates was performed, and it was demonstrated that KLK4 activates pro-KLK3 and cleaves members of the insulin-like growth factor binding protein family (IGFBP-3, -4, -5, and -6) [116]. KLK4 also activates pro-prostate specific antigen and degrades prostatic acid phosphatase [117]. It will activate meprin *β* [118] and urokinase-type plasminogen activator (uPA) [117] and also cleaves its receptor (uPAR) [119]. Recombinant human KLK4 mediates limited cleavage of types I and IV collagen, efficiently degrades the *α*-chain of fibrinogen [120] and was also shown to activate all pro-KLKs except for itself and KLK-7, -8 and -10 [121]. Additionally, KLK4 was proposed to have a signaling function via protease activated receptors (PARs) of the family of G protein coupled receptors, particularly PAR_1_ and PAR_2_ [122–125]. However, because during normal development (cancer excluded), the only apparent essential nonoverlapping function of KLK4 is in enamel formation, the substrate specificity of KLK4 is of little consequence unless those substrates are present during the transition to maturation stage of enamel development when KLK4 is normally expressed.

### 5.6. The Klk4 Knock-out/LacZ Knock-in Mouse

**Figure 3:**
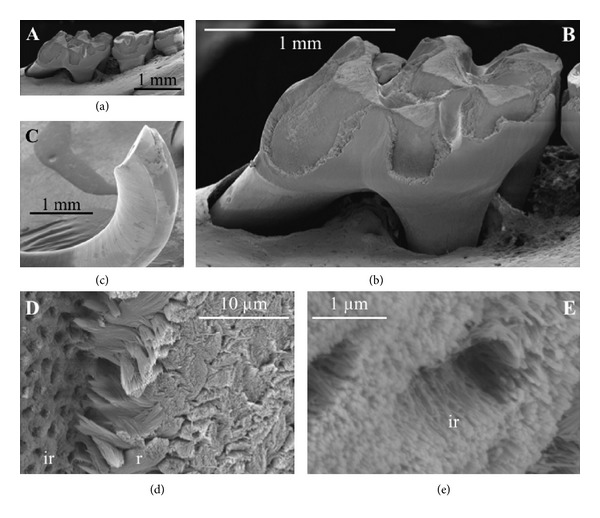
Scanning electron microscopy of the mandibular molars (a and b) and mandibular incisor (c–e) of a *Klk4 *null mouse at 7 weeks. The enamel of all molars showed a significant loss of enamel from all working surfaces (buccal cusps, occlusal surface, and marginal ridges) (a and b). Similarly, the enamel layer was abraded at the working (buccal) surface of the mandibular incisor at its tip (c). Higher magnification of the chipped area near the tip of the incisor showed that the break was in the enamel layer, close to, but not at the DEJ. The broken surface appears to be composed of interrod (*ir*) enamel with holes where enamel rods (*r*) had pulled out and separated (d) from the initial deposit of interrod enamel near the DEJ. The holes are too numerous to be made by odontoblastic processes penetrating the enamel (enamel spindles). The orientation of the crystallites on the walls of the holes is parallel to the direction of the tubular holes and to the crystallites between the holes (e). This figure was originally published in: Simmer et al. J. Biol Chem. 284 (28):19110-19121, 2009. The American Society for Biochemistry and Molecular Biology. DOI 10.1074/jbc. M109.013623.

The KLK4 preproenzyme is composed of 254 amino acids, while the proenzyme has 230 residues and the active form has 224 amino acids [47]. The *KLK4* genes of both mouse and human have 6 exons the first of which is noncoding. The mouse *Klk4* gene is approximately 10 kb in size and locates in cytogenic region B2 on mouse chromosome 7 [109]. Gene targeting was used to generate a mouse strain carrying a null allele of *Klk4* that has a nuclear *LacZ* reporter gene inserted directly into the *Klk4* translation initiation site. Therefore, the *LacZ *code was positioned in the same genomic context as wild-type *Klk4* and so provided a sensitive tissue reporter for native *Klk4* expression [15]. Other than a tooth phenotype, the *Klk4* ablated mice were normal. The teeth were normal, the enamel attained normal thickness, and no abnormalities were observed until the enamel reached the transition to early maturation stage of development. At this point, the normal export of enamel matrix proteins from the matrix back to the ameloblasts destined for lysosomal degradation was impeded. The enamel retained proteins that should have been removed and the soft, protein-rich enamel abraded from the mouse teeth (Figures 3(a)–3(c)). This strongly supports the supposition that KLK4 functions to cleave enamel matrix proteins to facilitate their export out of the hardening enamel [15]. However, the *Klk4 *ablated mice did reveal unexpected surprises. The rod enamel sometimes pulled away from interrod enamel as if the rod enamel were pegs on a cribbage board and the interrod enamel was defining the peg holes (Figures 3(d) and 3(e)). During the secretory stage, the ribbon-like enamel crystallites are surrounded by protein, but the approximate 10,000 to 40,000 crystallites that will interlock to form an enamel rod [126] are themselves surrounded by a tube-like protein layer (Felicitas Bidlack, personal communication). So, if this protein layer was not substantially removed, it can be envisioned that the rod and interrod enamel would not properly interlock with one another which would allow the “pegs to fall out of the holes”. Another surprise was that the individual crystallites themselves were prone to fall out of the rods. This was described as much like a circular bunch of “uncooked angel hair spaghetti” from which the individual strands fell. Although the normal rod pattern was present in the *Klk4 *ablated enamel, the 10,000 to 40,000 crystallites that the rod is composed of failed to interlock properly, and the crystallites fell from the rods [15]. Strikingly, the fact that the crystallites grew until they were expected to interlock with one another flies in the face of conventional theories of enamel formation. Conventional theories postulate that amelogenins inhibit growth in width and thickness of crystallites and that this growth will not occur until amelogenins are removed during the maturation stage of enamel development. In the *Klk4* ablated mice the amelogenins were not properly removed from the maturation stage enamel. Despite this, the crystallite ribbons grew into “spaghetti strands” thick enough to define an enamel rod and were almost ready to interlock with adjacent “spaghetti strands”. Therefore, amelogenins did not inhibit crystallite, growth in width and thickness by selectively binding to specific sides of the crystallites and consequently our “conventional theory” requires serious reexamination. 

More recent examination of the *Klk4* null mouse demonstrates that the outer enamel layer (most recently formed) is much harder than the inner enamel layers and that the enamel shows progressively less mineralization with depth [94, 95]. The reasons for this are unclear. Recall that odontoblasts do not express KLK4, so odontoblasts do not contribute to enamel matrix removal in the deep enamel layers during the maturation stage of development. Also recall that MMP20 activity is observed in maturation stage enamel from *Klk4* ablated mice. It was postulated that the continued MMP20 activity and endocytosis by ameloblasts combine to remove proteins from the surface enamel, but that KLK4 may be necessary to break up aggregates of accumulated enamel protein cleavage products in the deeper regions of the enamel layer so that they can return to the ameloblast for endocytosis [95]. All-in-all, the *Klk4* knock-in/knock-out mouse has revealed surprises about enamel formation and has forced us to reexamine some of our more firmly held beliefs about how crystallites grow in width and thickness to form an enamel rod. 

### 5.7. Human KLK4 Mutations

The human *KLK4* gene is located near the telomere of chromosome 19 (19q13.3-19q13.4) in a cluster of genes including the KLK family of serine proteases. Its gene exon/intron structure and protein domain structure are identical to those of the mouse [127]. A difference between the human KLK4 and KLK4 from mouse and pig is that human KLK4 has only one potential glycosylation site (Asn139) while the pig and mouse each have three potential glycosylation sites (pig: Asn104, Asn139, and Asn184; mouse: Asn93, Asn139, and 184). The reason for KLK4 glycosylation is incompletely known, but glycosylations can affect protein conformation, stability, and solubility, can protect against proteolysis, and can affect protein-protein and protein-mineral interactions. Native human KLK4 has never been isolated, but it was demonstrated that both pig and mouse KLK4 are variably glycosylated. Commercially available recombinant human KLK4 is not glycosylated and was shown to lose activity rapidly when compared to native pig and mouse KLK4. However, native pig and mouse KLK4 did lose activity when they were deglycosylated. So, it was deemed likely that glycosylation is important for KLK4 stability, presumably by protecting it from proteolytic degradation [128].

Two different human *KLK4* mutations are known to cause autosomal recessive hypomaturation AI. The first discovered is a nonsense mutation occurring upstream of the KLK4, catalytic domain (p.Trp153X). This tryptophan residue is completely conserved in mouse and pig KLK4 and expression of this mutated gene would result in a truncated protein lacking the final 101 amino acids which includes the catalytic triad (His71, Asp116 and Ser207). This homozygous mutation occurred in two female siblings, and both their primary and permanent dentitions were similarly affected. The sibling's teeth were yellow-brown in color and were excessively sensitive to hot and cold. The enamel was normal in thickness, but radiographically showed only a slight increase in opacity over that of the underling dentin indicating a decreased enamel mineral content. This soft enamel fractured from the occlusal surfaces of the primary molars [129]. No other phenotype resulted from this nonsense mutation in *KLK4*. The second human *KLK4* mutation was recently discovered by use of whole exome sequencing which identified a single nucleotide deletion (p.Gly82Alafs*87) in both alleles of a nine-year-old female. The frameshift was in the third of five coding exons, so the mutant *KLK4* transcripts may have been degraded by nonsense-mediated decay. If translated, the mutant protein would lack the same catalytic triad that was also lacking in the first discovered *KLK4 *mutation. As for the previously discovered KLK4 mutation, the enamel covering this proband's teeth appeared normal in size shape, but was discolored yellow-brown and chipped on multiple teeth. This proband was also secondarily affected with dental caries [103]. No other phenotype was observed due to the nucleotide deletion in *KLK4*. Therefore, both humans and mice have shown us that KLK4 is essential for enamel to achieve its final hardened form and that just as for MMP20, the only nonoverlapping function of KLK4 is in dental enamel development. 

## 6. Other Enamel Matrix Proteinases?

Human and mouse mutations in both *MMP20* and *KLK4* demonstrate that no other proteinase has an extensive overlapping function with either of these proteinases. If this was the case, no severe enamel phenotype would likely occur if the activity of MMP20 or KLK4 was compromised. However, in the past, prior to our knowledge of *MMP20* and *KLK4* mutations, we and others have proposed that various MMPs are present within the enamel matrix [62, 130–132]. Although some of the data are compelling, it is nonetheless difficult to reconcile that hydrolysis by MMP20 accounts for all the isolated amelogenin and ameloblastin cleavage products extracted from normal secretory stage porcine enamel [61, 66]. If another active MMP was in the enamel matrix, we would expect that amelogenin would be cleaved as has been demonstrated *in vitro *for at least MMP2 [132]. Also no enamel phenotype was demonstrated when these other MMPs were ablated from mice. Recently, MMP9 was proposed to be involved in controlling amelogenin processing and enamel formation [133]. These authors had *Mmp9* knockout mice, but failed to show an enamel phenotype for these mice. Chymotrypsin C (caldecrin, CTRC) was also recently shown as expressed in the enamel organ and was upregulated during the maturation stage of enamel development. The authors suggested that cathepsin C (CTSC) activates KLK4 which in turn activates CTRC [134]. However, although loss of CTRC function is a risk factor for pancreatitis, an associated enamel phenotype has not been described. Surprisingly, in mice lacking MMP20, a 40 kDa proteinase was observed by zymography of enamel extracts that were purified by reverse-phase high-performance liquid chromatography. Its presence was suggested to be a response by ameloblasts to the faulty array of inputs they receive from the defective extracellular matrix [114]. 

In all likelihood, most if not all of these proteinases are expressed in the enamel organ of forming teeth. The enamel organ is a dynamic structure that moves back to accommodate appositional mineral growth, and it also flattens as enamel matures. This moving and changing morphological structure would almost certainly require the enlistment of several active proteolytic enzymes. The difficult part is determining if these proteinases are secreted into the enamel matrix and are important for enamel formation. If any proteinase other than MMP20 and KLK4 function within the enamel matrix, it is likely they have overlapping functions with at least one other proteinase that precludes an enamel phenotype with loss of function. 

## 7. Enamel Matrix Proteins

### 7.1. Location and Function

The three major “structural” proteins in the enamel matrix of developing teeth are amelogenin, enamelin, and ameloblastin [135]. These proteins are derived from an ancestral gene belonging to the secretory calcium-binding phosphoprotein (SCCP) family [136]. Enamelin and ameloblastin map to a small area on the q arm of human chromosome 4 (4q13) [137]. The human amelogenin gene is on the X and Y chromosomes, but it was proposed that the amelogenin gene translocated to the sex chromosomes while enamelin and ameloblastin remained on their original chromosome [138–140]. Mutations in the X-chromosome amelogenin gene (*AMELX*) and mutations in the enamelin gene (*ENAM*) cause nonsyndromic enamel malformation (AI). Although disease causing mutations have not yet been observed in the human ameloblastin gene (*AMBN*), homozygous deletion of *exons 5 and 6* in the mouse *Ambn* gene results in almost no enamel formation [17, 141]. 

Two important points are worth highlighting about these enamel matrix proteins. First, they are necessary for proper enamel formation. However, they are later reabsorbed by the ameloblasts that originally secreted them into the matrix. So, these proteins are necessary for enamel formation, but they are not part of the final mature product. Only trace amounts of protein remain in mature enamel. Second, genes encoding these enamel matrix proteins have degenerated into pseudogenes in multiple toothless or enamelless species that descended from ancestors with teeth covered by enamel. Both birds and toothless baleen whales have degenerated amelogenin, ameloblastin, and enamelin genes [57, 142, 143], and a functional enamelin gene is absent in four different orders of placenta mammals that are toothless and/or enamelless [144]. This suggests that amelogenin, ameloblastin, and enamelin are only essential for dental enamel development and that there is no selective advantage in having these genes functionally maintained in species without enamel. Several groups have postulated that in addition to their role in enamel formation, specific enamel matrix proteins are important signaling molecules. If this was true, one would expect that these genes would remain functional in toothless and enamelless mammals due to the selection pressure of keeping the signaling pathways active. However, this is not the case.

### 7.2. Amelogenin

**Figure 4:**
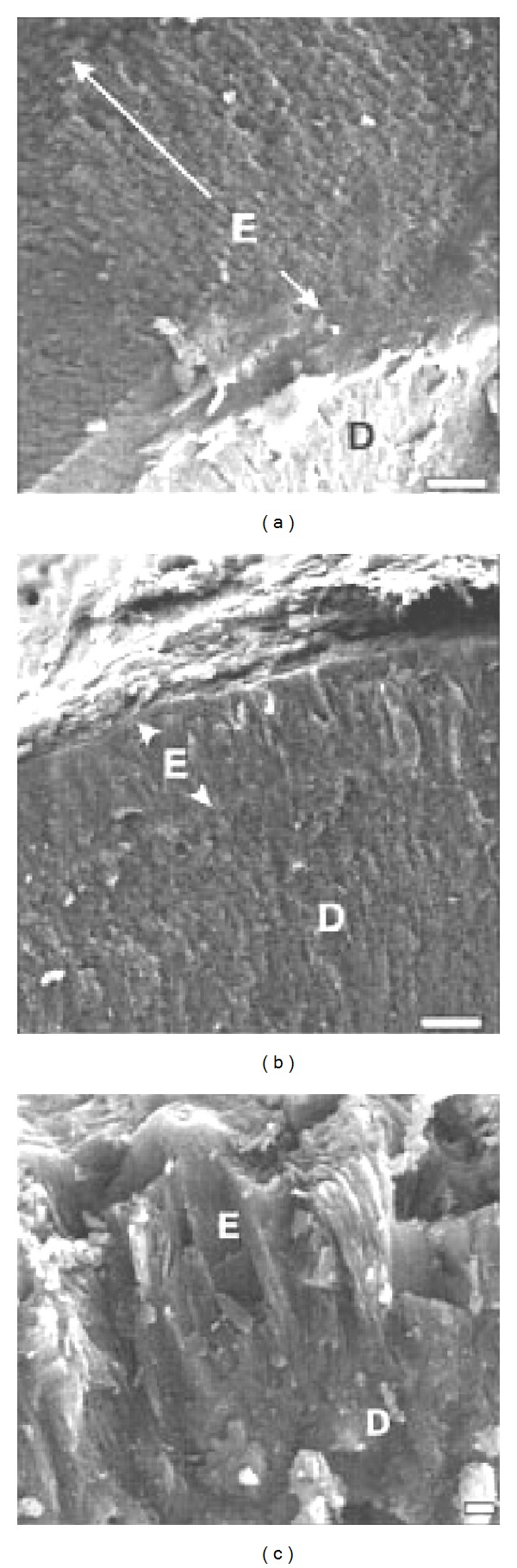
Scanning electron microscopy of fractured incisors from 16-week-old wild type and amelogenin null mice. The enamel (E) and junction with dentin (D) are shown. (a) wild type mouse. (b) the enamel from the *Amelx* null mouse does not have a normal prismatic structure and is markedly reduced in thickness compared with that of the wild type mouse shown at the same magnification as (a). (c) higher magnification of the enamel layer from the null mouse. *Arrowheads* indicate enamel thickness. Bars in (a) and (b) = 10 *μ*m; bar in (c) = 1 *μ*m. This figure was originally published in: Gibson et al J. Biol. Chem. 267 (34):31871-31875, 2001. The American Society for Biochemistry and Molecular Biology. DOI 10.1074/jbc.M104624200.

Amelogenin is the most abundant enamel matrix protein and it is essential for enamel formation [145–147]. Humans and pigs have two amelogenin genes each on the X-and Y-chromosomes while mice have just one on their X-chromosome [148]. The amelogenin amino acid sequence at its N- and C-termini is highly conserved among mammals, and the C-terminal region (13–15 residues) is highly charged (pI 4.2), whereas the entire protein has a pI of 8.0 [135]. In human males approximately 90% of amelogenin mRNA is expressed from the X-chromosome [149]. Mice with *Amelx* ablated from their genome have defective enamel that is hypoplastic and disorganized and that when fractured lacks a discernable enamel rod pattern (Figure 4) [16]. Amelogenin has only one posttranslational modification whereby Ser16 is phosphorylated [150, 151], but its transcripts undergo extensive alternative splicing [149, 152, 153] to generate at least 16 X-chromosomal murine amelogenin mRNAs [154–156]. The function of amelogenin alternative splicing is unclear. However, a recent study used transgenes to express the two most abundant *Amelx *transcripts in the amelogenin null mouse. These transcripts separately encoded the mouse 180 amino acid amelogenin protein (M180) and the mouse 59 amino acid protein (M59) termed leucine-rich amelogenin protein (LRAP). The amelogenin null mouse has enamel that is 10–20% of the usual thickness of wild-type enamel. The M180 but not the M59 transgene increased the enamel thickness and improved the rod pattern. However, when both transgenes were expressed in the same mouse, an improvement in enamel thickness and rod structure occurred over that of the M180 transgene alone. This enamel was a bit more than half the thickness of the wild-type molar enamel and was only about one third the thickness of wild-type incisor enamel [157]. Therefore, although the reasons for the alternatively spliced amelogenin transcripts remain unclear, they do appear to contribute to the formation of fully thick enamel with a proper decussating enamel rod pattern. 

Interestingly, numerous *in vitro *studies have addressed the question of how amelogenin promotes enamel formation. Since amelogenin is the most abundant enamel matrix protein, these studies made sense when little was known about the other matrix proteins that are much less abundant. Amelogenin is also relatively easy to express and purify and unlike ameloblastin which is glycosylated and enamelin which is highly glycosylated, amelogenin has only one posttranslational modification consisting of a single phosphate. Therefore, amelogenin can be purified in bacterial expression systems or, due to its abundance, purified from immature pig teeth. However, neither of these purification schemes is feasible for the mass purification of ameloblastin and enamelin necessary for the large posttranslationally modified quantities required to perform *in vitro* crystal growth experiments. Currently, we know from mouse knock-out/knock-in studies that amelogenin by itself cannot initiate crystal growth or promote enamel formation. When the enamelin [18] or ameloblastin [17] gene is ablated from mice, these mice have no true enamel and no crystal structure. Strikingly, the amelogenin null mice have measurable crystals with a well defined organization. The crystals are much smaller and less well organized than in wild-type mice, but they are nonetheless present [158]. It is unfortunate that ameloblastin and enamelin are so difficult to isolate and purify because we now know from knock-out/knock-in mice just how essential they are for enamel development. 

To date, eighteen *AMELX* mutations including two complete gene deletions [159] were shown to cause human X-linked AI. X-linked AI accounts for about 5% of all AI cases [160]. The enamel phenotype varies with the location of the mutation, but a unique aspect of certain *AMELX *mutations is that affected women may have vertically ridged teeth with alternating bands of normal and hypoplastic enamel. This is presumably due to ameloblasts that have randomly inactivated either the normal or defective X-chromosomes during development [161]. Affected males have a more severe phenotype. No AI cases have been reported from mutations in *AMELY* and two individuals with *AMELY, *deletions had normal teeth [162]. Although *AMELX* and *AMELY *each contain seven exons, these genes have diverged and do not undergo homologous recombination [163]. This is why they are used in forensics for sex determination. Furthermore, since the *AMELY *gene does not function in enamel formation or have any other known function, no positive selection exits for its maintenance and forensic analyses have demonstrated that *AMELY* is frequently deleted from the Y-chromosome [164]. It is worth noting that like amelogenin ablated mice, deletion of the amelogenin gene (*AMELX*) in humans does result in a thin enamel layer. This layer is the thickest on the cusp tips and marginal ridges relative to the lateral tooth surfaces [159]. It appears that amelogenin is not necessary for the nucleation of enamel crystallites, but is necessary for the crystallites to continue to grow in length in an organized manner.

### 7.3. Ameloblastin

Ameloblastin is the second most abundant enamel matrix protein. The *AMBN *gene locates to chromosome 4q21 and has 13 exons [165, 166]. Ameloblastin was first described as a nonamelogenin protein extracted from pig that migrated between 13 and 17 kDa on SDS-PAGE [167]. Immunohistochemical experiments showed that this protein locates between the enamel rods in an area termed the sheath space for which the proteins were therefore named sheath proteins [12]. The proteins locating to the sheath space are ameloblastin cleavage products. In contrast, intact ameloblastin, in the outermost newly formed enamel, accumulates on the enamel rods and not in the sheath space as do the cleavage products [168, 169]. This was the first data suggesting that full-length ameloblastin performs one function at the mineralizing front and that it performs a different function once it is cleaved and accumulates in the sheath space [10]. 

At approximately the same time, three groups independently cloned ameloblastin cDNAs from rat (two groups) and from pig. Therefore, three different names were proposed. The rat protein was named “ameloblastin” [165] and “amelin” [170], and the pig protein was named “sheathlin” [171]. The gene name has been designated “ameloblastin” (*Ambn*). In pig, the intact secreted ameloblastin protein is composed of 395 amino acids, but migrated on SDS-PAGE at a higher than expected molecular mass of approximately 62 kDa. Subsequently, It was discovered that ameloblastin is O-glycosylated at Ser86 [172] and Thr348 [173], hydroxylated at Pro11 [171] and Pro324 [173], and has four serines (Ser15, Ser17, Ser209, and Ser210) in the required context for phosphorylation by Golgi casein kinase [174]. The ameloblastin mRNA is alternatively spliced, and splicing determines the glycosylation state of the ameloblastin protein [172]. Excluding the signal peptide, the translation products are 380 and 395 amino acids, and the smaller ameloblastin isoform differs from the larger by the absence of 15 amino acids encoded at the 5′ end of exon 5. Although its function remains unknown, this N-terminal region is highly conserved among species and contains the Ser86 O-glycosylation site [172]. Therefore, ameloblastin undergoes several posttranslational modifications and is alternatively spliced, and translation of the smaller splice product results in the loss of a highly conserved domain that contains one of only two ameloblastin O-linked phosphorylation sites. 

Intact ameloblastin is a trace component of developing enamel and has never been isolated *in vivo* [68]. As mentioned previously, cleavage of ameloblastin by MMP20 accounts for all the known ameloblastin cleavage products in the porcine enamel matrix. The initial cleavages release three products from the N-terminal region which include the 17 and 13 kDa products originally observed on SDS-PAGE gels [167] and also include a 15 kDa cleavage product [175]. A subsequent comprehensive analysis of MMP20 ameloblastin cleavage site preferences suggested that MMP20 initially cleaves the large ameloblastin splice product (395 residues) at one of three sites near the N-terminus (after Gln130, Arg170, or Ala222) to generate cleavage products. These initial products are then cleaved a second or third time at these same sites as well as at specific secondary sites that are located mostly near the C-terminus [68]. The N-terminal cleavage products accumulate in the sheath space throughout the enamel layer while the calcium binding C-terminal cleavage products are on the rods and are not detectable beyond a depth of 50 *μ*m from the surface of the newly formed enamel [168]. This is consistent with the proposed 3D human ameloblastin model demonstrating that ameloblastin has N- and C-terminal domains connected by an unstructured linker that is susceptible to degradation [176]. Therefore, this reinforces previous data by showing that ameloblastin N- and C-terminal cleavage products locate to different areas of the forming enamel and likely play different roles in enamel development. 

Ameloblastin is a pseudogene in certain species of baleen whales [57] and, although published reports suggest critical functions in other tissues, deletion of *Ambn *from mice appears to only affect enamel development. A recent publication suggested that ameloblastin is important in root development. However, the report of the original ameloblastin mutated mouse showing a severe enamel phenotype stated that: “…root formation in the mutant mice was not different from wild-type and *Ambn* ^+/−^ mice” [17]. Perhaps the most published alternative function for ameloblastin is in bone remodeling and repair. However, if the NCBI UniGene database is accessed and AMBN is used as a search term, the resulting EST Profile demonstrates that not a single *AMBN *transcript has been found in either human or mouse bone. Conversely, the mouse but not the human EST Profile contains an EST analysis from molar and *Ambn *transcripts were found in mouse molar tissues. The evidence for ameloblastin expression in bone may be conclusively confirmed or denied by the soon to be published characterization of the newly engineered *Ambn-LacZ*-knock-in mouse that substitutes *LacZ *into, and *Ambn *out of, the natural *Ambn* translational start site (J.P. Simmer: personal communication).

**Figure 5:**
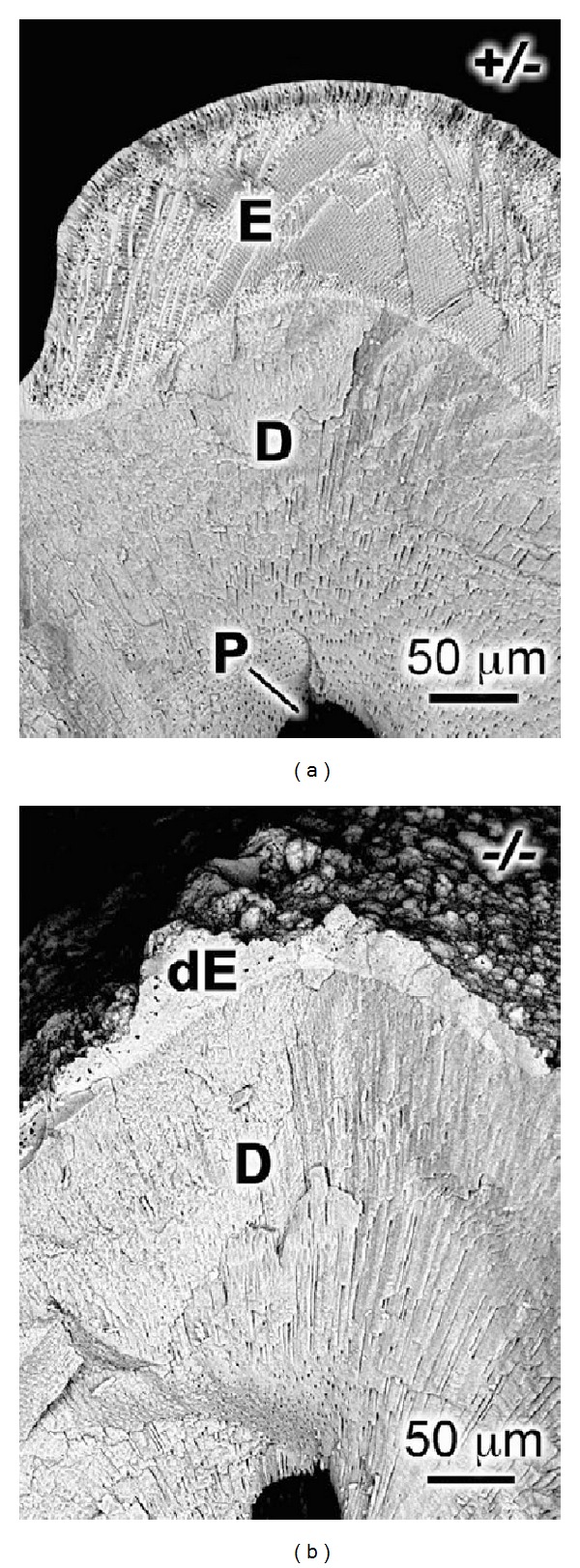
Defects in enamel formation of ameloblastin null mice. SEM analysis of 8-wk-old incisor cross sections from heterozygote and homozygote mice. Note the lack of a true enamel layer on the homozygous mutated *Ambn *incisor. E, enamel; D, dentin; P, pulp. (D) SEM analysis of incisor. E, enamel; D, dentin; P, pulp; dE, defective enamel. This figure was originally published in: Fukumoto et al. J. Cell Biol. 167 (5):973-983, 2004. D01/10.1083/jcb.200409077.

Although human mutations in *AMBN *that cause AI have not yet been discovered, a mouse model exists where exons 5 and 6 are deleted from *Ambn*. Originally this mouse was deemed as a true *Ambn* knockout [17], but subsequently it was discovered that in these same mice an *Ambn* mRNA lacking exons 5 and 6 was expressed and that this truncated mRNA was also translated in cells from mouse enamel organ [141]. However, as mentioned previously, the mutated protein causes a severe enamel phenotype where a very thin layer of dysplastic mineralized material is deposited on the dentin, and this material has no rods no crystals and does not resemble enamel (Figure 5) [17, 141]. Interestingly, during the presecretory stage, when the ameloblasts are attached to a basement membrane adjacent to where the enamel will form, the *Ambn* mutant ameloblasts are normally arranged and start their characteristic process of elongation into secretory ameloblasts. However, after the early secretory stage, when the basement membrane is destroyed, these same ameloblasts abnormally detach from the matrix layer, lose their cell polarity, and appeared to fold over one another to form multilayered cell structures [17]. This was attributed to ameloblastin playing a role in cell adhesion. However, ablation of the enamelin gene results in a near identical progressive dysplastic ameloblast layer morphology as is observed in the *Ambn *mutated mouse [177]. An alternative explanation for the dysplastic ameloblast layer morphology may be that since no enamel layer forms in the *Ambn* mutated and *Enam *ablated mice, ameloblasts could fail to adhere to the unnatural surface even if their attachment apparatus remained intact. Once the basement membrane is degraded, ameloblasts no longer have a tight binding site adjacent to the mineral surface and as they progress into the secretory stage, the ameloblasts normally adhere weakly to the enamel surface. Furthermore, wild-type ameloblasts move back as the enamel layer rapidly thickens and cell proliferation at the cervical loop compensates for this movement of ameloblasts away from the dentin. So, when enamel thickening is absent, as in the case with *Ambn* and *Enam* mutations, the ameloblasts occupy a smaller surface than normal that could contribute to their dysplastic morphology [177]. Although the *Ambn* mutant mice have an ameloblast layer morphology that is disrupted after the early secretory stage, this may be a secondary effect from the almost complete absence of the rapidly thickening enamel layer. Ameloblastin and enamelin likely play a more central role in crystallite nucleation and subsequent crystallite elongation. 

### 7.4. Enamelin

The human enamelin gene (*ENAM*) spans 18 kb on chromosome 4q11-21, and its transcript has nine exons with a single noncoding exon 1 [178]. The human enamelin mRNA encodes a preprotein of 1142 amino acids, and no alternatively spliced enamelin mRNAs have ever been identified [69]. The enamelin protein is secreted as a 186 kDa precursor phosphorylated glycoprotein which quickly undergoes a series of proteolytic cleavages [127, 179, 180]. The secreted 186 kDa (amino acids 1–1104) porcine parent protein can only be found within 1 *μ*m of the enamel surface [70]. This protein is rapidly processed from its C-terminus to generate 155, 145, and 89 kDa N-terminal cleavage products that are short lived and are only found near the enamel surface. The 32 kDa enamelin cleavage product (amino acids 136–241) is the only stable domain that accumulates in the deeper enamel [70]. This 32 kDa enamelin proteolytic fragment is highly conserved among species. The three N-linked glycosylation sites originally described in the porcine 32 kDa enamelin were unchanged during mammalian evolution [178]. This suggests that the 32 kDa enamelin cleavage product plays an important functional role in enamel formation. Furthermore, among mammals, sites of enamelin posttranslational modifications are highly conserved. This includes several potential and known phosphorylation and N-linked glycosylation sites and includes six cysteines that are thought to form disulphide bridges [142]. Therefore, enamelin is a large extensively post-translationally modified protein that is highly conserved among mammals and undergoes proteolysis soon after its secretion. Like amelogenin, ameloblastin, and MMP20, enamelin function is only required in mammals that have enamel on their teeth. In mammals without enamel or without teeth, enamelin may become a pseudogene [57]. 

**Figure 6:**
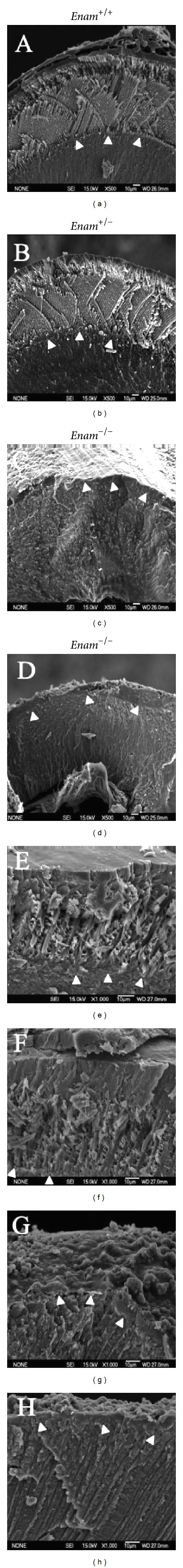
Scanning electron microscopy of wild-type, heterozygous, and enamelin null mouse enamel from incisors and molars at 7 weeks. SEM was used to examine fractured sections of mouse incisors (a–d) and molars (e–h). The enamel of wild type (a and e) and heterozygous (b and f) both showed a thick enamel layer with well-defined rod (prism) structures. In contrast, the enamel of *Enam *null mice (c, d, g, and h) was extremely thin and irregular, with a rough surface. In some places (g), the enamel did not even form sufficiently to complete the DEJ. *Arrowheads *delineate the DEJ. This figure was originally published in: Hu et al. J. Biol. Chem. 283 (16):10858-10871, 2008 by The American Society for Biochemistry and Molecular Biology. DOI/ 10.1074/jbc.M710565200.

Mutations in the mouse enamelin gene were originally induced with the mutagen N-ethyl-N-nitrosourea and four separate point mutations were identified. These were p.Ser55Ile, p.Glu57Gly, a T to A substitution at the splice donor site in exon 4, and p.Gln176X. The heterozygous mouse phenotypes included a rough and pitted enamel surface and the homozygous mutant mice had enamel agenesis [181, 182]. Since then, as was performed for KLK4 (see Section 5.6.), an *Enam* knock-out/*LacZ* knock-in mouse line was developed. Gene targeting was used to generate a knock-in mouse carrying a null allele of *Enam* that has a *LacZ* reporter gene replacing the *Enam* translation initiation site and gene sequences through exon 7 [18]. Thus, the *LacZ* reporter construct was in the precise genomic location that *Enam* had been knocked out of and was therefore transcriptionally regulated in precisely the same way as the *Enam* gene. *LacZ *expression provided a sensitive reporter for native *Enam *expression and the knock-in mice showed *LacZ* expression only in the ameloblasts of developing teeth. In addition, teeth from *Enam* ^−/−^ mice lacked enamel and had a white opaque appearance with severe dentin abrasion along the labial and lingual sides of the teeth. The molars from these mice had pronounced occlusal wear so that the cusp tips were flattened and rounded. The erupted portions of the incisors had very thin enamel that was coated with a layer of small calcified material that: “felt gritty and sandpaper-like in consistency” (Figure 6). *μ*CT reconstructions confirmed the lack of mineralized enamel in the molar crown and incisor crown from *Enam* ^−/−^ mice. Strikingly, the secretory stage enamel layer of *Enam* ^−/−^ mice was von Kossa negative except for small mineralized nodules that were more abundant near the dentin-enamel junction. The mandibular incisors from *Enam* ^+/−^ mice were consistently chalky white, whereas the maxillary incisors were variable ranging from near normal to chalky white. The mandibular incisors were also subject to occlusal wear, and the functional incisal edge of the incisor was always missing. Also, the enamel from mandibular incisors of *Enam* ^+/−^ mice had a mineral to protein ratio that was normal during the secretory stage, but in the early maturation to nearly mature stages this ratio was half that of wild-type mice [18, 183]. A thick layer of enamel protein accumulates in the extracellular space beneath the secretory ameloblasts in the *Enam* ^−/−^ mice, but no mineralization occurs at the mineralization front. Therefore, enamelin is thought to be a critical component of the mineralization front that promotes or catalyzes the extension of enamel crystallites [18]. Both enamelin and ameloblastin appear to have similar functions with regard to crystallite initiation and elongation, whereas amelogenin appears to create a framework that allows the continued elongation of the already initiated crystallites. 

Mutations in *ENAM *cause AI. The first reported AI-causing mutation in *ENAM* was a heterozygous G to A transition in the first nucleotide of intron 8 that was predicted to cause a deletion of exon 8 (p.A158-Q178del) during mRNA processing [184]. The tooth crowns were small, thin, and yellow with little or no enamel. When a single *Enam *allele is defective, the phenotype can be nonpenetrant [185] or be manifested as enamel pits [186], horizontal groves [187], or generalized thin enamel [184]. When both *ENAM* alleles are defective, the enamel is extremely thin or nonexistnt [186]. In most cases, however, *ENAM* mutations cause autosomal dominant AI. To date, twelve novel *ENAM* disease-associated mutations have been characterized two of which are caused by two different mutations in each *ENAM* allele (compound heterozygosity). The compound herterozygous mutations were discovered in a family where the proband and his father had an AG insertion (p.422FsX448) in *ENAM* previously identified in AI kindreds from Slovenia and Turkey, whereas the mother and proband had a novel missense mutation that substitutes leucine for a phosphorylated serine (p.Ser216Leu) in the 32 kDa enamelin cleavage product [188]. The proband had discolored small teeth consistent with hypoplastic AI. Both the father and the mother had teeth that appeared highly polished with localized pitting defects. The most recently discovered *ENAM* mutation identified a novel heterozygous frameshift mutation in exon 4 (p.Asn36Ilefs56). This frameshift occurs in the coding region for the signal peptide which is predicted to preclude synthesis of the entire secreted protein. Both the proband and his father had thin, soft enamel, and the incisal edges of their primary anterior teeth were chipped [189]. Therefore, enamelin is the largest, least abundant, and most highly post-translationally modified enamel matrix protein. It is essential for enamel development but may become a pseudogene in enamelless or toothless mammals [144].

## 8. Summary and Perspective

This review on “Dental Enamel Development: Proteinases and their Enamel Matrix Substrates” has thus far provided a mostly fact-based update on the proteins known to be present within the enamel matrix. In this section, these facts will be woven into a plausible mechanism by which these enamel matrix proteins support and promote enamel development. The new facts support a somewhat different interpretation of enamel development than what was previously accepted. It must be stated that although the proteins of the enamel matrix (amelogenin, ameloblastin, enamelin, and MMP20, KLK4) are essential for enamel formation because the malfunction of any one of them causes AI, they are not the only proteins that are essential. Mutations in *FAM83H* [190] and *LAMB3* [191] cause autosomal dominant AI, and mutations in *WRD72* [192], *C4orf26* [193], and *SCLA24A4* [194] cause autosomal recessive AI. Several other gene mutations cause defective enamel as part of a syndrome [103]. However, the proposed mechanism of enamel development focuses on just what may occur within the enamel matrix itself so that the enamel can develop and ultimately achieve its final hardened form.

Recall that each enamel rod is composed of approximately 10,000 to 40,000 crystallites and that each crystallite starts in the shape of a ribbon (about 26 nm by 68 nm in cross-section) [126]. Previously, it was widely accepted that amelogenin serves to inhibit crystallite growth in width and thickness as the crystallites extend from the dentin-enamel junction to the surface of the tooth. MMP20 was and still is proposed to cleave enamel matrix proteins so that the cleavage products will locate to different areas of the forming enamel to support the elongation of the enamel crystallites. KLK4 was proposed to cleave the enamel matrix proteins, principally amelogenin, to facilitate protein removal and to allow the crystallites to grow in width and thickness. However, from the results of the *Klk4* ablated mice [15], we now know that amelogenin does not completely inhibit crystallite growth in width and thickness. Ablation of *Klk4* in mice resulted in enamel with a higher protein content [94] because the proteins were not efficiently removed from the enamel matrix. However, despite this, the enamel crystallites grew substantially in width and thickness. The crystallites did not interlock and actually spilled out of the rods as what appeared to be “angel hair spaghetti”. It is possible that the crystallites grew to the point where they could interlock, but the excessive protein present may have prevented this. Regardless, the presence of amelogenin still allowed the crystallites to grow substantially in width and thickness to the point where they could almost interlock. 

Conversely, as described in Section 7.2, amelogenin by itself cannot initiate crystal growth or promote enamel formation. When the enamelin [18] or ameloblastin [17] gene is ablated from mice, these mice have no true enamel and no crystal structure. However, the amelogenin null mice have measurable crystals with a well-defined organization. The crystals are much smaller and less well organized than in wild-type mice, but they are nonetheless present [158]. Therefore, amelogenin does not inhibit crystallite growth in width and thickness by binding selectively to specific sides of the crystallites and it does not initiate crystallite growth. So, what is the most abundant enamel matrix protein doing to promote enamel development? A significant clue comes from the finding that newly formed enamel ribbons start as amorphous calcium phosphate (ACP) that then transform into hydroxyapatite (HAP) [195, 196]. This means that the enamel ribbons are established prior to their crystallization. The size shape and spatial organization of the enamel crystallites must be set when the ACP is first formed so that it can crystallize into the proper ribbon shape. Therefore, amelogenin is proposed to form a mold that sets the boundaries for the crystallite ribbons. Much as cement is poured into a mold for a house foundation, ACP would be poured in or establish itself within the confines of the amelogenin ribbon mold. Although the 32 kDa enamelin cleavage product that is highly conserved among mammals and that persists in the enamel matrix has not been shown to bind with amelogenin *in vivo*, it was shown to colocalize with amelogenin [197]. Thus, it is likely that the 32 kDa enamelin cleavage product is also a structural component of the postulated amelogenin ribbon molds. Along this same line of reasoning, it would also make sense that the ameloblastin MMP20 cleavage products that accumulate in the enamel sheath space may also provide a structural component for the mold. Certainly, the mold must be adequately supportive so that the long thin ribbons do not break as they grow in length. 

So, it is possible that full-length enamelin and ameloblastin and/or their C-terminal cleavage products promote the growth of the future crystallites in length. This makes sense because the full-length proteins and/or their C-terminal cleavage products are only present at the mineralization front and are not present in the older, deeper enamel layers. Subsequently, and almost immediately, these proteins and amelogenin are cleaved by MMP20, and some of these N-terminal cleavage products accumulate within the enamel to form a mold that establishes the shape, size, and orientation of the forming crystallites. Once the end of the secretory stage is attained and the ribbons stop growing in length, KLK4 is secreted to cleave the enamel matrix protein mold so that it can be exported from the hardening enamel so that the maturing crystallites can interlock and form a cohesive rod structure. 

This new theory [126] represents a departure from previous beliefs that amelogenin by itself initiates enamel formation and from the thought that amelogenin inhibits crystallite growth in width and thickness. Results from genetically altered mice clearly demonstrate that neither of these widely held beliefs is true. The newly proposed theory incorporates the results obtained from genetically altered mice and is therefore based on what occurs *in vivo* during enamel development. Theories are only the closest approximate of the truth that we have at this point in time. Future theories, if they are to be taken seriously, will have to be consistent with results generated from studies performed *in vivo*. After all, it is nature that guides us to its mysteries.
